# Evaluating a pilot eLearning program as an educational tool for child health and developmental surveillance in general practice

**DOI:** 10.15694/mep.2020.000255.1

**Published:** 2020-11-12

**Authors:** Jonathan Nguyen, Natalie Ong

**Affiliations:** 1 Royal Darwin Hospital; 2 The Children's Hospital at Westmead

**Keywords:** Medical Education, Primary Care, General Practice, Child Health, Child Development, eLearning

## Abstract

This article was migrated. The article was marked as recommended.

**BACKGROUND AND OBJECTIVE:** To evaluate a pilot eLearning program (Sydney Local Health District Well Child Health program) and whether it could improve the knowledge of general practitioners and trainees in child health and developmental surveillance.

**METHOD:** A mixed method quantitative and qualitative study was performed involving comparisons of pre and post-course test results, and analysis of feedback surveys following a face-to-face general practice training workshop along with in-depth telephone interviews.

**RESULTS:** Comparisons of pre and post-test scores of 25 course participants demonstrated a significant mean score increase of 2.92 out of 14.00 (CI 1.56-4.28; *P* < 0.001). Qualitative results from 18 feedback surveys and 8 telephone interviews provided mostly positive feedback regarding the information content and usefulness in meeting educational needs.

**DISCUSSION:** With increased uptake and completion, the Sydney Local Health District Well Child Health eLearning program has the potential to be an effective medical education tool for general practitioners and can lead to an increase in knowledge of child health and developmental surveillance.

## Introduction

The early identification of child health and developmental concerns, and subsequent intervention, has shown to lead to positive outcomes later in life, optimising developmental skills and improving health outcomes especially in children diagnosed with developmental and behavioural issues ( Shonkoff and Hauser-Cram, 1987; Anderson *et al.*, 2003; Guevara *et al.*, 2013). In Australia, primary health care for children is provided by general practitioners (GPs) in conjunction with child and family health nurses, with GPs often being the first point of call if concern arises for a child’s health. Consequently, GPs are well placed to provide surveillance and screening for child health and developmental issues through child health checks. In the state of New South Wales (NSW), the child’s Personal Health Record (cPHR) is a valuable screening tool developed by the state government that can be used as an adjunct to surveillance and screening in child health and development ( *Child Personal Health Record (Blue Book) Release of Revised Version 2012/2013*, 2013). The cPHR has high rates of use by parents and carers across the state, although often not completed properly ( *2007-2008 Report on Child Health from the New South Wales Population Health Survey*, 2010). Primary care practitioners can provide this guidance, however receive no formal training themselves, and so the cPHR is often underutilised in everyday consultations with children ( Ong *et al.*, 2013). Deficiencies have since been identified in surveillance for behavioural conditions ( Garg *et al.*, 2015; Woolfenden *et al.*, 2015).

Community Paediatric Services of a health district in metropolitan Sydney developed the ‘Well Child Project’ (WCP) which was launched in 2017. The WCP encompasses face-to-face teaching sessions and a web-based eLearning tool to upskill GPs in navigating the cPHR and to perform more comprehensive child health and developmental surveillance in everyday practice. The anticipated benefits of the project were to increase awareness and competency of GPs in performing child health and developmental surveillance. As a result, there would be potential for earlier, less missed diagnoses and timely referrals for intervention. This would improve a child’s developmental trajectory, flowing on to positive long-term outcomes into adulthood.

### The Well Child Health eLearning program

The Well Child Health program is a free online resource hosted within the health district’s website ( https://www.slhd.nsw.gov.au/WellChildHealth). It is an educational tool which takes the participant through five interactive core modules covering child health and developmental surveillance:

1. Examination of the infant & young child
2. Hearing, vision and growth
3. Anticipatory guidance topics
4. Child development & surveillance
5. Putting it all together

Participants complete a pre-course test prior to commencing the five modules. In each module they are provided with background information and links, with a series of multiple-choice questions and case studies to complete at the end. Following each question, the participant is provided with the correct answer with an explanation and hyperlinks to more references and information. At the end of each module, a total score and percentage of correct answers is shown. The five modules can be completed in any order although sequentially from one to five is preferred. Those who complete all five modules and pass the post-course test (80% success required) receive a certificate of completion and ability to claim continuing medical education points from the Royal Australian College of General Practitioners.

## Methods

### Study Aims

The purpose of the study was to evaluate the Well Child Health eLearning program. The primary objective was to assess whether completion of the eLearning resulted in the improvement of knowledge of primary health practitioners in child health and developmental surveillance. The secondary objective was to gather information on the strengths, weaknesses, and areas for improvement for the eLearning program.

### Methods

A mixed method analysis of data was performed. Data on the eLearning program was collected and evaluated through three primary methods:

• Quantitative data obtained directly from the eLearning program’s inbuilt pre and post-course tests

The Well Child Health eLearning program was advertised through a local NSW GP training program and via word of mouth. Participants were able to login and undertake the eLearning modules at their own leisure. Study participants provided online consent for collection of data prior to commencing the eLearning.

The eLearning module incorporated a pre and post-course test consisting of the same 14 multiple choice questions. Following completion of the pre-course test, participants were given their score but not the answers. Following completion of the 5 modules, participants completed the same test (post-course) and were provided with their scores and answers. Data from the pre and post- course test were examined to identify if there was any improvement of scores following completion of the 5 modules.

• Mixed data obtained from participant feedback surveys following face-to-face training workshops in Child Development run by a GP registrar training organisation

Workshop participants were sent an email before the event asking them to complete the eLearning module prior to attendance. Following the workshop, an email survey was sent to all workshop participants to gather feedback about their workshop experience including the pre-activity eLearning program. The survey consisted of Likert scale style questions asking participants to grade the eLearning program from several perspectives, and open-ended questions regarding highlights and areas for improvement. Free-text responses were included in analysis.

• Qualitative data obtained through in-depth telephone interviews with eLearning participants

All GP registrars who had participated in the 2018 face-to-face workshop were invited to participate in further feedback sessions regarding the eLearning program. GP registrars who consented were contacted to determine if they were happy to participate in a telephone interview. Interviewees were asked about their participation in the eLearning program from several perspectives; baseline demographic information was obtained, as well as questions covering the technical aspects of the online modules in addition to their experience of each section of the modules and whether it had affected their everyday practice. The interviews were recorded and transcribed.

### Ethics

The study was approved by the Sydney Local Health District’s ethics review committee.

### Statistical Analysis

Data was analysed using SPSS Statistics v25 (IBM Corp., Armonk, NY, USA). As no previous data on pre and post-test score differences existed, a retrospective power calculation was performed to determine that the number of participants and mean difference in pre and post-test primary outcome scores would be sufficient to generate at least 80% power and *P* value of ≤ 0.05. Continuous data was tested for normal distribution and subsequently analysed using a two-sided paired *t*-test.

## Results/Analysis

### 1. eLearning data:

Data from the eLearning program itself was collected from December 2017 to September 2019. There were 170 general practitioners and registrars who completed the initial eLearning registration of which 128 were GP registrars and 42 were GPs. Of these, 165 participated in the activities of module one, 94 in module two, 100 in module three, 72 in module four and 55 in module five. Twenty-five participants completed the eLearning through to the post-course test after completing the five modules, with the number of days to completion ranging from 0 to 125 with a mean of 14 days.

**Figure 1.**
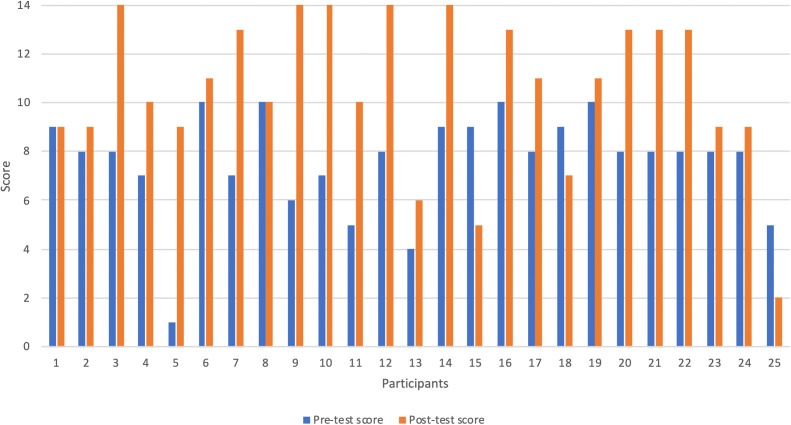
Pre and Post-test scores

A retrospective power analysis demonstrated that the 25 participants would be sufficient to detect the mean difference of 2.92 in pre and post-test scores with 98.7% power and *P* < 0.05. The pre and post-course quiz results of the 25 participants revealed a mean pre-course test score of 7.60 out of 14.00 and mean post-course test score of 10.52 out of 14.00. There was a significant increase of 2.92 in the mean score following completion of the eLearning modules (CI 1.56-4.28; *P* < 0.001) ( Figure 1).

### 2. Workshop surveys:

One hundred and fifty-six GP registrars attended the November 2018 workshop with 85 completing the workshop evaluation survey. Of these, 18 had undertaken the eLearning and were able to provide feedback. Depending on the question, answers were ranked from 1 to 10 and 1 to 5.

The eLearning was scored favourably with higher mean scores indicating that participants agreed the eLearning was informative, engaging and practical ( Table 1). Higher mean scores also showed that participants found the eLearning useful in meeting learning needs with clear content clarity ( Table 2). Examples of participants’ responses are shown in Box 1 and 2.

**Table 1.** Workshop evaluation survey 1

	Number of GP Registrar responders	Mean score
Informative	11	8.36
Engaging	12	7.17
Practical	16	7.31

1 = strongly disagree and 10 = strongly agree

**Table 2.** Workshop evaluation survey 2

	Number of GP registrar responders	Mean score
Meeting learning needs	17	4.12
Content clarity	17	4.06

1 = not useful/clear and 5 = very useful/clear

**Table:** 

Box 1: Examples of participant’s comments on ‘what worked well?’ in the eLearning: • ‘Clear guidelines and reasoning.’ • ‘Good [information] on preventive care, growth and development and on resources to provide to parents. Video on how to examine an infant was helpful as well.’ • ‘Useful practical information.’ • ‘Well structured, covered common issues, in-built quizzes.’ • ‘[The eLearning] covered topics and [provided] extra reading if needed.’ • ‘Videos and autism app.’

**Table:** 

Box 2: Examples of participant’s comments on ‘what could be improved?’ in the eLearning: • ‘Quizzes had poor correlation with content presented, very frustrating at times when the questions were unanswerable unless you had specific knowledge.’ • ‘Information was quite dense on the page, would be better if the chunks were broken up a bit more.’ • ‘More specific information, clearer redirection to resources, better alignment between content and quizzes (some disparity between content and answers given, and some questions asked on content on covered).’ • ‘Interface a bit clunky on mobile.’

### 3. Telephone interviews:

Forty-nine GP registrars who had participated in the face-to-face programs gave consent to be contacted for further feedback, with eight subsequently participating in a telephone interview. Most participants were between age 26 to 34 and were in their second to third year of GP training. All reported very little previous exposure to child health surveillance, including those who had completed paediatric hospital terms and the University of Sydney’s (USYD) Sydney Child Health Program. The reported number of paediatric patients seen daily ranged from 5 to 40, and all reported to have used the cPHR. It took participants two to ten hours to complete the eLearning with most completing in around three hours.

#### Technical aspects of the eLearning:

Most participants reported no significant technical issues, with the eLearning described as ‘easy to navigate’ with a ‘good layout’. Two participants mentioned difficulty signing up and running the eLearning on certain browsers and technological platforms such as smartphones. Three participants reported difficulties with the quizzes including losing answers if idle for too long or being unable to save answers. Most participants would have liked a ‘next’ button when moving between pages.

#### Pre and post-course assessments:

There were no significant issues with the assessments. Feedback included finding the assessments difficult, and the question formats of ‘ordering from most to least correct’ or ‘selecting more than one correct answer’ being a deterrent. One user mentioned minor factual inconsistencies with the eLearning content and assessment answers provided.

#### The five eLearning modules:

All participants agreed that the content of the five modules was interesting and applicable. Content was described as ‘practical’, and ‘well-structured and referenced’. Many participants mentioned the ‘Child Development & Surveillance’ module as the most helpful. Highlights included practical tips provided through videos, especially in the ‘Examination of the infant & young child and ‘Hearing, vision and growth’ modules, as well as case studies within each module. Two participants mentioned excessive content and links to click through. Two mentioned the eLearning as a concise resource they would refer back to in the future. Examples of participants’ comments are shown in Box 3.

**Table:** 

Box 3: Examples of participants’ comments on the content of the five modules: • ‘Excellent. I think it’s perfect for GPs. So much information, especially the first couple of modules and anticipatory guidance, exactly what we do day to day, so it’s really, really good.’ • ‘I really liked the case studies because it’s ... a reallife example of what parents might circle on the blue book as exploring what they’re worried about and then how do you address it after that. And the model answers were pretty good.’

#### The eLearning and the face-to-face workshop:

All participants suggested that eLearning was best completed before the face-to-face workshop and complemented the teaching of the workshop. Most suggested introducing the eLearning as early as possible in training.

#### Suggestions to improve:

Participants were overall satisfied with content, however suggested for more information to be provided on behavioural problems, referral pathways, immunisation counselling and infant feeding. They suggested having the eLearning available earlier in the GP training pathway with more advertising and incentives required to ensure uptake of the program.

#### Translation to real-world practice:

All participants reported a positive change in practice including more confidence with child health checks, a better structured approach to child development through examination and documentation, and being better able to advise parents through anticipatory guidance. Examples of participants’ comments are shown in Box 4.

**Table:** 

Box 4: Examples of participants’ comments regarding how eLearning experience has changed everyday practice: • ‘It’s helped with my examination technique when I see children...I also want to use the blue book a bit more as a communication tool.’ • ‘[I’m] more confident firstly in examining the child appropriately, screening developmentally and also giving appropriate advice to parents about the anticipatory guidelines.’ • ‘I think the anticipatory guidance [information] changed the most in my consultations.’ • ‘A lot, yes like maybe 70%. I think a baby check is very important and also the documentation of Blue Book and recording, as well as explanation[s] to the parents.’ • ‘I think that [my] knowledge is probably a lot better and just having that better awareness of what I’m looking for. [The eLearning] sort of compliments [USYD’s Sydney Child Health Program] a lot more as well, so it’s been good.’ • [I’m] more competent with the referral pathway about what’s benign and what’s something to actually refer.’

## Discussion

The results of this study support the potential effectiveness of this pilot eLearning program as an educational tool in child health and developmental surveillance. Comparison of 25 participants’ pre and post-test scores revealed a significant mean increase of 21% in scores. This demonstrates that the completion of the eLearning modules can lead to an increase in knowledge of the participants. Qualitative results obtained through telephone interviews also support this finding with an increase in participants’ self-perceived competency in consultations reflecting a gain in knowledge.

The secondary objective to gather feedback on the eLearning program itself revealed that participant satisfaction was high, especially with the information content provided and its applicability. The videos demonstrating examples of child health checks were highlighted multiple times which proves their strength as an engaging learning tool. There was a strong preference towards the ‘Child Development and Surveillance’ module which may reveal that development and behavioural issues are key areas of child health that GPs find difficult to navigate. More concentration in this area, particularly in including content on referral pathways could be considered in future revisions. Encouragingly there was feedback that the eLearning module not only strongly complemented the face-to-face workshops, but also could complement other child-health related education such as USYD’s Sydney Child Health Program. Comments that participants would refer to the eLearning resource in future practice also demonstrated the program’s potential as a valuable long-term source of education.

The authors however acknowledge the significant limitations of this evaluation, particularly the low proportion of participants completing the eLearning through to the post-course test. Personal time constraints, pending examinations and lack of incentives to complete the program likely played a role. Feedback also revealed that there were multiple system issues (such as glitches) of the pilot program on different technological platforms and internet browsers, which interfered with the user experience and possibly deterred participants from continuing. In terms of the knowledge gain, participants completing the eLearning over a longer time period may have had the benefit of exposure to additional educational experiences, as well as the ‘learning effect’ where the program may have influenced participants to seek other related education. The use of a control group to complete both pre and post-course tests without exposure to the eLearning modules would have helped measure the extent of the learning effect.

The potential of eLearning for continuing medical education in a primary healthcare setting has been received positively by GPs ( Gensichen *et al.*, 2009). It enables access to continuing education opportunities with the flexibility of completing at one’s own time and pace, without geographical distance limitations. For a busy clinician, this allows the education to be more adaptive to their personal learning needs ( Ruiz, Mintzer and Leipzig, 2006). This study is the first of our knowledge that evaluates an eLearning system which provides education in child health and developmental surveillance. It adds to the growing evidence that eLearning is an innovative tool in providing medical education, with similar studies of eLearning programs in the literature also demonstrating their effectiveness in a variety of medical education settings ( O’Leary and Janson, 2010; Rudolf *et al.*, 2010; Hemans-Henry, Greene and Koppaka, 2012). Importantly eLearning does not have to substitute traditional instructor-led training but can form part of a blended-learning strategy to effectively provide continuing medical education. This is supported by our telephone interview results where participants stated that participating in the eLearning prior to the face-to-face workshop was complementary to consolidating their learning.

## Conclusion

The SLHD Well Child Health eLearning program has the potential to be an effective medical education tool for child health and developmental surveillance for NSW general practitioners. With ongoing improvements/revisions of the pilot program and incentives for increased uptake and completion, it may have a larger positive impact in the primary care setting. Further studies are required to not only examine the role of this eLearning within a blended-learning strategy, but also to explore the effects of such educational interventions on long-term child health outcomes.

## Take Home Messages

• Early identification of children with health and developmental concerns leads to better productivity and quality of life for children and their families.
• General practitioners in Australia are often the first point of call in providing surveillance and screening for child health and developmental issues through child health checks.
• The SLHD Well Child Health eLearning program has the potential to be an effective medical education tool in child health and developmental surveillance for GPs and GP registrars in NSW.

## Notes On Contributors

**Jonathan Nguyen** is an advanced trainee in general paediatrics at Royal Darwin Hospital, Darwin, Australia. Jonathan has an interest in primary health care and education and has completed a Masters in International Public Health. ORCID ID: https://orcid.org/0000-0001-5282-6902

**Natalie Ong** is a developmental paediatrician from the Children’s Hospital Westmead Clinical School, Sydney, Australia with experience conducting training for health professionals in child health and development. Natalie has completed her Masters in Clinical Education and is pursuing her PhD in improving care for children with developmental disabilities in hospital. ORCID ID: https://orcid.org/0000-0002-0962-443X
