# Supplementary material for: Evaluating a pilot eLearning program as an educational tool for child health and developmental surveillance in general practice
Source: MedEdPublish (2016). 2020 Nov 12;9:255. [Version 1] doi: 10.15694/mep.2020.000255.1 (PMC10697438; doi:10.15694/mep.2020.000255.1)
Supplement: Peer review history

# Reviewer report: Reviewer response for version 1

**DOI:** 10.21956/mep.20162.r31155

**Published:** 2021-01-30

**Authors:** Ken Masters

**Affiliations:** 1 Sultan Qaboos University

This review has been migrated. The reviewer awarded 3 stars out of 5 A nice and straight-forward piece of research reporting on an evaluation of a pilot eLearning program as an educational tool for child health and developmental surveillance in general practice.Overall, most of the information has been well-presented. There are, however, some areas that need to be addressed:• More information about the theming of the qualitative data is required (who themed, checked, was software used, etc).• A copy of the questionnaire. I can understand that the authors do not wish to share the test questions, but the questionnaire questions should be shared.• Similarly, the telephone interview questions.• Readers would like to see parts of the course. At the moment, the online course requires registration, and people might not be keen to supply all those details just to see the contents of the course. As a result, it would be useful to supply some screenshots or some have a sample of the course in an area that does not require registration. • When reporting results (response rates included), please supply percentages as well as raw numbers.• The methods say that baseline demographics were obtained (in the telephone interviews), but these are only vaguely reported (e.g. “Most participants were between age 26 to 34…”) . Please can these be reported in more detail.• In addition, were there demographic data in the survey form? If not, why not? If so, please report the results.• It would also be useful if the chief results could be reported in these demographic sub-groups to see if there are any statistically significant differences.• The small sample size really does have to be listed as a limitation.I look forward to Version 2 of the paper in which these issues are addressed.

Reviewer Expertise:

NA

No decision status is available

# Reviewer report: Reviewer response for version 1

**DOI:** 10.21956/mep.20162.r31154

**Published:** 2020-12-18

**Authors:** Barbara Jennings

**Affiliations:** 1 Norwich Medical School

This review has been migrated. The reviewer awarded 4 stars out of 5 I will strongly recommend this article to educators who are interested in paediatrics; community medicine; and postgraduate clinical education.The rationale for the intervention, to improve the early identification of child health and developmental concerns, by increasing the knowledge and skills of general practitioners is explained clearly and justified in the introduction and discussion.The study evaluated a well-constructed educational intervention in a rigorous way. The methodology was presented clearly, and the authors provide links to the website hosting the e-package and the governance and data analysis was all robust and explained fully.The quantitative results about learning gain and the qualitative analyses are presented well and any limitations for the study (re sample size and generalisability) are discussed.The medical education community has never been in greater need of e-learning packages with established validity and utility. This research will have contributed to a thorough evaluation of the New South Wales child health e-package, which no doubt has been developed further and well used in 2020.

Reviewer Expertise:

NA

No decision status is available
